# 1235. Roles of Tetracyclines for Treatment of *Stenotrophomonas maltophilia* Pneumonia

**DOI:** 10.1093/ofid/ofab466.1427

**Published:** 2021-12-04

**Authors:** Taha Alhayani, Carolyn Philpott, Siyun Liao, Anthony Gentene, Eric Mueller

**Affiliations:** 1 Trihealth Good Samaritan Hospital, Cincinnati, Ohio; 2 UC Health, Cincinnati, Ohio; 3 UC Health-University of Cincinnati Medical Center, Cincinnati, OH

## Abstract

**Background:**

*Stenotrophomonas maltophilia* is a multidrug resistant organism with limited antibiotic treatment options. Sulfamethoxazole-trimethoprim (TMP-SMZ) is considered first line agent based on *in vitro* studies and clinical evidence. Minocycline has been showed to be active on *in vitro* studies and also has been explored in small retrospective studies However, doxycycline in the same class has variable in susceptibility in *in vitro* studies and has not been evaluated for efficacy in treatment of *S. maltophilia* infections The purpose of this research is to compare minocycline and doxycycline to TMP-SMZ for treatment of *S. maltophilia* pneumonia.

**Methods:**

This retrospective, multi-center study evaluated hospitalized patients treated for *S. maltophilia* pneumonia with minocycline, doxycycline, or TMP-SMZ for clinical success, microbiologic success, and recurrence or reinfection within 30 days that required treatment. The inclusion criteria were patients ≥18 years old with *S. maltophilia* confirmed on respiratory culture from January 2013 to November 2020. Patients were classified as treatment with tetracyclines (minocycline or doxycycline) or TMP-SMZ based on definitive agent used for ≥50% of the treatment course and a minimum of four days. Patients with *S. maltophilia* resistant or intermediate to definitive therapy, and patients with combination therapy for treatment for *S. maltophilia* pneumonia were excluded.

**Results:**

A total of 21 patients were included in tetracyclines group and 59 patients included in TMP-SMZ group. There was no difference in clinical success (28.6% vs. 25.4%; *P* = 0.994) or microbiologic success (n=28, 55.6% vs. 66.4%; *P*= 0.677) between tetracyclines and TMP-SMZ, respectively. Recurrence or reinfection requiring treatment (n=24) was higher in the tetracyclines group but not statistically significant compared to TMP-SMZ (66.7% vs. 26.7%; *P*= 0.092). A subgroup analysis showed no difference between doxycycline, minocycline, and TMP-SMZ for these three aims.

**Conclusion:**

Clinical and microbiologic success were similar in patients treated with tetracyclines compared to TMP-SMZ for *S. maltophilia* pneumonia. This data suggests minocycline and doxycycline may be an option to treat *S. maltophilia* pneumonia, but conclusive clinical data continues to be lacking.

**Disclosures:**

**Anthony Gentene, PharmD**, **advisory board with Theravance Biopharma and Mylan** (Consultant)

